# MemBrain: Improving the Accuracy of Predicting Transmembrane Helices

**DOI:** 10.1371/journal.pone.0002399

**Published:** 2008-06-11

**Authors:** Hongbin Shen, James J. Chou

**Affiliations:** Department of Biological Chemistry and Molecular Pharmacology, Harvard Medical School, Boston, Massachusetts, United States of America; University of Queensland, Australia

## Abstract

Prediction of transmembrane helices (TMH) in α helical membrane proteins provides valuable information about the protein topology when the high resolution structures are not available. Many predictors have been developed based on either amino acid hydrophobicity scale or pure statistical approaches. While these predictors perform reasonably well in identifying the number of TMHs in a protein, they are generally inaccurate in predicting the ends of TMHs, or TMHs of unusual length. To improve the accuracy of TMH detection, we developed a machine-learning based predictor, MemBrain, which integrates a number of modern bioinformatics approaches including sequence representation by multiple sequence alignment matrix, the optimized evidence-theoretic K-nearest neighbor prediction algorithm, fusion of multiple prediction window sizes, and classification by dynamic threshold. MemBrain demonstrates an overall improvement of about 20% in prediction accuracy, particularly, in predicting the ends of TMHs and TMHs that are shorter than 15 residues. It also has the capability to detect N-terminal signal peptides. The MemBrain predictor is a useful sequence-based analysis tool for functional and structural characterization of helical membrane proteins; it is freely available at http://chou.med.harvard.edu/bioinf/MemBrain/.

## Introduction

### Motivation for a more accurate TMH predictor

Membrane-embedded α helical, polytopic proteins constitute the majority of ion channels, transporters, and receptors in living organisms. This class of proteins, which account for ∼40% of all membrane proteins, are difficult targets for high resolution structural studies. Although experimentally determined structures of integral membrane proteins have been increasing at a fast rate in recent years, they only sum to less than 1% of the structures in the Protein Data Bank (PDB). Probably the first analysis that researchers perform when studying a helical membrane protein, whether it is for functional or structural characterization, is prediction of TMHs from the protein amino acid sequence. Knowledge of TMHs is very useful in initial elucidation of the overall topology of the protein, as well as in the rational design of protein constructs for structural studies.

Computational tools for TMH prediction are widely available. In this paper and in previous papers on TMH prediction, TMH is defined as a segment of helix that is embedded in the membrane. Hence, TMH sequence ends when the transmembrane region ends, although the helix can continue beyond the membrane. In general, residues of TMHs are mostly hydrophobic. Hence, earlier TMH prediction programs, such as TOP-PRED [1], compute sequence hydrophobicity from amino acid hydrophobicity scales assigned by biophysical and chemical measurements [2]–[4], and predict TMH propensity based on the average hydrophobicity score of a sliding prediction window of *N* successive residues along the sequence. Later predictors use more statistics-based, machine learning techniques. For example, PHDhtm [5] is based on neural networks, and TMHMM [6] and Phobius [7] are based on the hidden Markov model. The available TMH predictors are used routinely in membrane protein characterization and, in most cases, are sufficiently reliable in providing descriptive information about TMHs [8].

However, as more high resolution structures of helical membrane proteins become available, we learn that TMH has a wide length distribution. About 5% of the TMHs in the known structures are very short (<15 residues) and only span the membrane partially. These helices are known as the ‘half TMHs’ (see an example in the structure of the glycerol-conducting channel [9]). Very long TMHs (>40 residues) have also been found in the membrane proteins, e.g., the metalloenzyme particulate methane monooxygenase protein [10]. None of the existing TMH predictors perform satisfactorily in detecting TMHs of irregular lengths. For example, TOP-PRED [1] predicts all the TMHs to be 21 residues long, TMHMM [6] cannot predict TMHs shorter than 16 residues or longer than 35 residues, and SOSUI [11] cannot predict TMHs longer than 25 residues.

We developed a TMH prediction method, named MemBrain, which aims to improve the accuracy of TMH prediction. MemBrain was trained using the standard training dataset that was used by many other predictors, yet performed ∼20% better than others when tested with a benchmark testing dataset. The improvement came mainly from the capability of MemBrain to predict accurately the ends of TMHs and therefore to detect TMHs of irregular lengths. Such capability was realized by applying the powerful **o**ptimized **e**vidence-**t**heoretic **K**-**n**earest **n**eighbor (OET-KNN) prediction algorithm [12]–[14] to protein sequence representations that include sequence evolution information, and by merging results from prediction sequence windows of different sizes. Our results show that, with the fast expanding database of experimental membrane protein structures, there is still much room for improving the accuracy of TMH prediction using a pure statistics-based protocol.

## Results

### The algorithm

**Figure 1:**
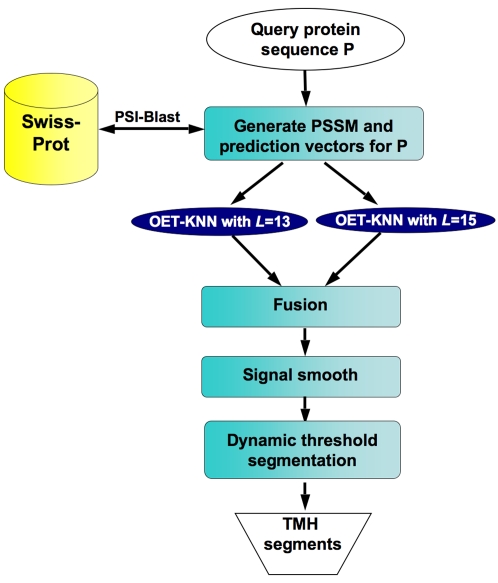
A flowchart diagram of the MemBrain protocol.

A flowchart of the MemBrain predictor is shown in Figure 1. We represented a protein sequence of *N* residues by the **p**osition-**s**pecific **s**coring **m**atrix (PSSM) (*N* rows and 20 columns), generated using the PSI-BLAST program [15] (see Methods section). The PSSM contains sequence evolution information from multiple sequence alignment against the SWISS-PROT protein database, and therefore provides a more complete description of the characteristics of a protein sequence. The propensity of a residue at positions *i* for being a part of a TMH was predicted based on a sequence segment of length *L* centered on *i*, where *L* is an odd number that represents the prediction window size. The prediction window size has a profound effect on the prediction outcome. Large window size, e.g., *L* = 17 (used in the PHDhtm predictor [5]), is more effective for predicting residues in the middle of a long TMH due to higher content of neighborhood information. However, it performs poorly for residues near the ends of TMHs, and is incapable of predicting half TMHs shorter than 15 residues. On the other hand, if *L* is too small, the prediction accuracy generally suffers as a result of losing the neighborhood sequence information. In the MemBrain predictor, we combined two window sizes to minimize the bias caused by the use of only one window size. We found that the fusion of two window sizes, 13 and 15, gave the best prediction results.

For TMH prediction, we used the standard training dataset which was used by most other TMH predictors, including TMHMM [6], Phobius [7], THUMBU [16] and SVMtm [17]. This dataset includes 50 helical membrane proteins of known TMH regions (see Supplementary Table S1). For each of the 50 proteins, the PSSM was generated using the PSI-BLAST program. From the PSSM, the matrix elements (*L*×20) for various sequence segments of *L* = 13 or 15 were extracted and stored in the training vectors 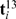 or 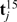, respectively (see Methods section for details of constructing these vectors). These training vectors were labeled as ‘TMH’ if the residue *j* at the middle of the sequence segment is a part of a TMH, and were otherwise labeled as ‘NOT TMH’. From the 50 PSSMs, we built a training set of 14,531 vectors of *L* = 13 and 14,431 vectors of *L* = 15. These vectors were used as statistical rulers for making predictions on the target protein.

Given a query protein, the PSSM was constructed and the query vector for sequence segment centered on residue *i* (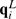) was defined. To predict the TMH propensity of residue *i*, denoted here as **E** *_i_*, we applied the OET-KNN algorithm for which the inputs are the query vector 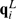 and all 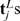 in the training set with the same dimension. The OET-KNN algorithm is a classification tool which has proven to be powerful in pattern recognition [12], [14] as well as in the prediction of sub-cellular locations of proteins [13], [18]. In the OET-KNN calculation (described in details in the Methods section), the Euclidean distances between 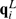 and all 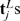 were calculated, and the 50 closest matches were used to calculate **E** *_i_*, which ranges from 0 to 1, where 0 and 1 are zero and unity probability of TMH, respectively. The TMH propensity obtained for *L* = 13, 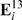, was merged with that obtained for *L* = 15, 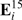, by simple averaging. Thus the combined TMH propensity for residue *i* is 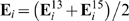, ranging from 0 to 1. The procedure was repeated to cover all residues, (*L*-1)/2≤*i*≤*N* – (*L*-1)/2, in the query protein.

For a query protein, the E*_i_* vs. *i* plot gives an overview of the residue-specific TMH propensity. We used the median filter technique [19] to smooth the TMH propensity profile, and at the same time, to reduce noise. The final step is to determine the TMHs based on the smoothened propensity profile. In most other predictors, a fixed threshold is used to segment the scores, i.e., residues having scores larger than the threshold are assigned as TMH [11], [17], [20]. However, the optimal threshold for defining two TMHs separated by long loops is very different from the threshold required for identifying TMHs separated by short loops or tight turns. High-resolution structures show that two consecutive TMHs are often connected by very short loops or turns. In these cases, since the loop residues only represent a small region of the prediction window, the TMH propensity calculated for the short loops are higher than those of long loops. To solve this problem, we used a dynamic threshold method in which a base threshold propensity of 0.4 was first used to define TMH fragments. Then we raised the threshold according to the shape of the local propensity profile for identifying short loops or helical breaks in these fragments (see Methods section for details).

Finally, in some membrane proteins, the first N-terminal TMH is a N-terminal signal peptide. We included an extra module in the MemBrain program to detect potential N-terminal signal peptide in a membrane protein using methods described in ref. [21].

### Performance

To test the MemBrain predictor and compare its performance with the existing TMH predictors, we constructed a testing dataset consisting of 70 helical membrane proteins of known high resolution structures which do not overlap with the training dataset (see Supplementary Table S2). There are a total of 378 TMHs in the testing dataset. The performances of the TMH predictors were evaluated with four different scores.

1. *The TMH prediction success rate (V_TMH_)*. V_TMH_ is simply the fraction of TMHs in the testing set that are correctly predicted [22]; it is defined as
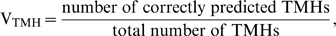
(1)where a TMH is considered predicted correctly if it has an overlap of at least 9 residues with the prediction. However, we note that such definition is not robust, and in some other studies, different lengths of residue overlap were used [22], [23].
2. *The protein prediction success rate (V_P_)*. V_P_ is the fraction of helical proteins in the testing set that are correctly predicted [22]; it is defined as
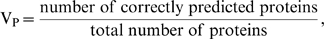
(2)where a protein is considered predicted correctly if all the TMHs in this protein are correctly predicted (as defined in V_TMH_ above) and the number of predicted TMHs is equal to the observed number of TMHs in the protein.
3. *The N and C scores*. These two scores evaluate the accuracy of predicting the ends of TMHs [22]. N and C scores are the number of N- and C-terminal residues that do not match when aligning the predicted and observed TMHs. In the best case, if the predicted and observed TMHs are completely matched, the N and C scores equal to 0.
4. *The normalized RMSD*. Finally, we calculated the normalized distance between the predicted and known TMH representation vectors, denoted by **p** = [*p*_1_, *p*_2_,…, *p_N_*], in which *p_i_* is assigned to 1 if residue *i* is a part of a TMH and is otherwise assigned to 0. The normalized distance, or RMSD*_N_*, is defined as
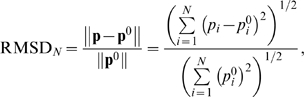
(3)where **p** and **p** ^0^ are the predicted and known TMH representation vectors of a protein, respectively. The normalized RMSD is less subjective than the definition of V_TMH_ and V_P_ above.

Table 1 compares the performances of MemBrain and other TMH predictors as judged by the four different scorings described above. MemBrain performs significantly better than other predictors in all four scoring categories. The V_TMH_ and V_P_ scores have been widely used in evaluation of TMH predictors. MemBrain V_TMH_ and V_P_ scores are 97.9% and 87.1%, respectively, which are about 6–16% better than Phobius (the best performer in this scoring category among the published predictors). MemBrain also has an improved capability to predict correctly the ends of TMHs as shown by the mean N and C scores of 3.2 and 3.1, which are about 20% better than the best published predictor for this scoring category. Finally the MemBrain mean normalized rmsd is 0.35, also about 20% better than the second-best performing predictor Phobius. The observed and predicted TMHs for the 70 membrane proteins in the testing dataset are given in Supplementary Data S1.

**Table 1:** Performance comparison of various TMH predictors^a^.

Predictor	V_TMH_	V_P_	N-score	C-score	RMSD*_N_*
THUMBU[16] b	85.5%	47.1%	6.9±4.9	6.7±4.9	0.58±0.19
SOSUI[11] c	89.1%	57.1%	5.0±4.1	5.0±4.2	0.44±0.21
DAS-TMfilter[20] d	90.7%	64.3%	6.5±5.0	5.5±5.3	0.58±0.16
TOP-PRED[1] e	92.6%	60.0%	4.5±3.8	4.6±3.9	0.45±0.15
TMHMM[6] f	91.0%	65.7%	4.5±3.8	4.5±3.9	0.44±0.15
Phobius[7] g	91.8%	71.4%	4.6±4.0	4.4±4.1	0.44±0.19
**MemBrain** h	**97.9%**	**87.1%**	**3.2**±3.0	**3.1**±2.8	**0.35±0.14**

a The testing dataset consists of 378 TMH segments from 70 proteins (see Supplementary Table S2).

b http://sparks.informatics.iupui.edu/Softwares-Services_files/thumbup.htm [16].

c http://bp.nuap.nagoya-u.ac.jp/sosui/ [11].

d http://mendel.imp.ac.at/sat/DAS/DAS.html [20].

e http://bioweb.pasteur.fr/seqanal/interfaces/toppred.html [1].

f http://www.cbs.dtu.dk/services/TMHMM/ [6].

g http://phobius.cgb.ki.se/ [7].

h http://chou.med.harvard.edu/bioinf/MemBrain/.

## Discussion

**Figure 2:**
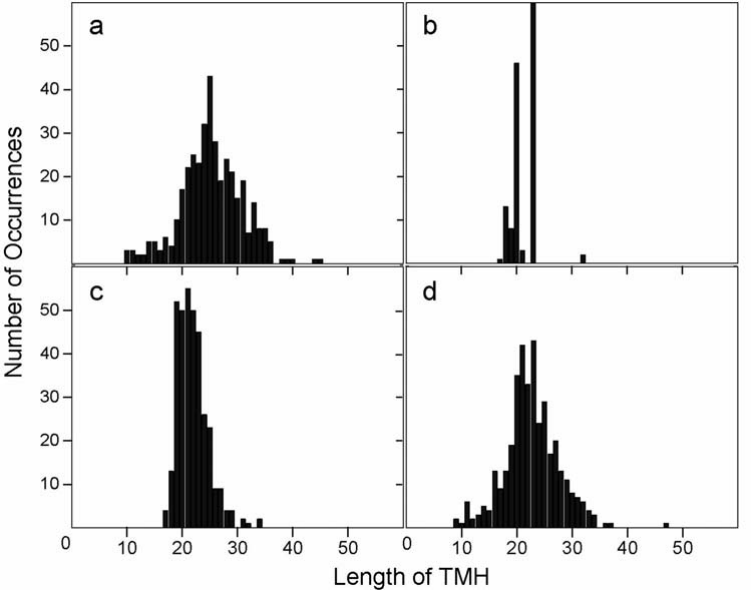
TMH length distribution in (a) 70 known membrane protein structures in the testing dataset, (b) TMHs predicted by TMHMM [6], (c) TMHs predicted by Phobius [7], and (d) TMHs predicted by MemBrain.

The above prediction scores obtained from a fairly complete testing dataset show that MemBrain is the best TMH predictor to date. Probably the most attractive feature of MemBrain is the improved ability in correctly identifying the ends of TMHs. This capability is important because there is a wide distribution of TMH length amongst the 70 helical polytopic membrane proteins in the testing dataset (Fig. 2a), e.g., TMH can be as short as 10 residues. Most TMH predictors cannot detect TMHs shorter than 15 residues (e.g., Figures 2b&c show that the shortest TMH predicted by TMHMM and Phobius, the predictors which gave the second best N and C scores in Table 1, is 17 residues). However the length distribution of TMHs predicted by MemBrain matches most closely to that of the observed dataset (Fig. 2d). We also noticed that MemBrain shows similar improvements in prediction when considering only TMHs that are longer than 15 residues (see Supplementary Table S3).

The improvement came from a combination of the steps used in our protocol shown in Figure 1. First, the PSSM representation contains sequence evolution information, which provides more complete sampling for statistical prediction methods. The advantage of a pure statistical approach over hydrophobicity-based prediction methods is that the prediction outcome does not depend on our interpretation of amino acid sequence in TMH formation, which could introduce bias. Second, the OET-KNN algorithm is a powerful classification method that can combine many different evidences and deal with the uncertainty to reach the optimal decision. Third, the fusion of two prediction window sizes provides more flexibility in accounting for length variation of TMHs, and thus reduces the bias towards a fixed TMH length introduced by using only one window size (as treated in all the previous predictors). Finally, assignment of TMHs using the dynamic threshold method further refines the prediction by detecting short loops and turns that separate TMHs.

A somewhat unsatisfying aspect of the TMH-only prediction is the complete absence of amphipathic, extramembrane helices that are common in helical membrane protein structures. In both the training and testing datasets, the TMH sequences are defined to end when the transmembrane regions end. However, according to many high resolution structures, a considerable portion of transmembrane helices extend well beyond the lipid bilayer and become hydrophilic. Therefore, TMH predictors cannot predict the extramembrane portions of helices. Our future direction is to develop methods to predict both transmembrane and extramembrane helical segments in helical polytopic membrane proteins.

## Methods

### Construction of query and training vectors

The PSSM matrix of a protein **P** of *N* residues, which contains sequence evolution information, is defined as
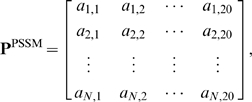
(4)where *a_i,j_* denotes the probability of residue *i* of the protein being changed to amino acid type *j* as determined from multiple sequence alignments [15]. The matrix elements in Eq. 4 were generated using the PSI-BLAST [15], which searches the SWISS-PROT database (version 52.0 released on 6-March-2007) against the sequence of the protein. For prediction studies, a residue at position *i* of the protein can be represented by a query vector, 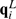, composed of the PSSM matrix elements of the query protein corresponding to a sequence segment of length *L* centered on *i*, e.g.,
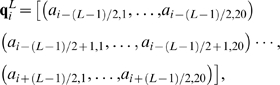
(5)where *L* is an odd number. Eq. 5 is also used to construct training vectors, 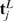, from their corresponding PSSM matrices of proteins in the training dataset.

### Calculation of TMH propensity

Consider the problem of predicting the propensity of residue *i* of the query protein belonging to a structural pattern, denoted by φ, where
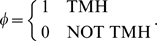
(6)We represent the residue by a query vector 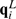 (see Eq. 5 above), constructed for prediction window size *L*. The knowledge basis used for the prediction is given by the training dataset, T*^L^*, e.g.,
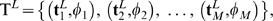
(7)where vectors 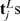 were also constructed as in Eq. 5 for window size *L*, and their corresponding patterns φ*_j_*'s are known.

Let S_K_ be a set of vectors consisting of K 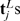 in T*^L^* that have the shortest Euclidean distances to 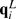, referred to here as the K nearest neighbors of 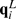. For any 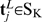, the knowledge that 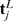 has a pattern φ is a piece of evidence which increases our belief that 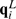 also has the pattern φ. This evidence is quantified, as in refs. [24], [25], by an *evidence function*

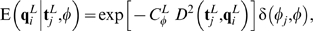
(8)where 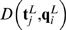 is the Euclidean distance between 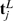 and 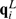, and the parameter 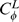 is associated with a particular pattern φ; the delta function in Eq. 8 is
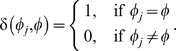
(9)In OET-KNN, 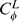 is optimized by maximizing the prediction accuracy of every sample in T*^L^*. Using the detailed optimization protocol described in ref. [14], we found the following values of 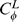: 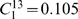, 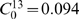, 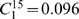, and 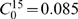.

Combining the knowledge of the K nearest neighbors in S_K_, the *evidence* of 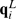 belonging to the pattern φ is
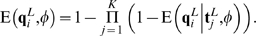
(10)The final *evidences* 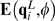 are then normalized as in
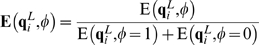
(11)to satisfy the normalization condition 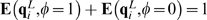.

Finally, after merging the prediction results obtained using two different window sizes, *L* = 13 and 15, the propensity of residue *i* belonging to TMH is
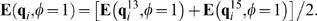
(12)


### Dynamic threshold segmentation

**Figure 3:**
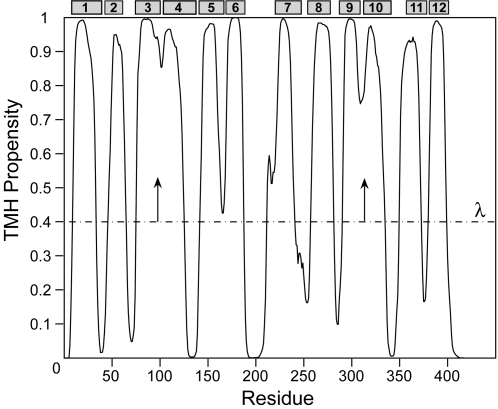
The residue-specific TMH propensity of lactose permease of *Escherichia coli* (PDB code: 1PV7) [26], illustrating the method of assignment of TMHs by dynamic threshold segmentation. The observed TMHs, assigned in ref. [26], are shown as the gray boxes.

To assign TMH fragments based on the propensity profile, we used a dynamic threshold segmentation approach. First, residues with propensity greater than or equal to 0.4 were considered as TMH. The base threshold, λ = 0.4, was selected by optimizing the self-consistency test performance as was done in refs. [11], [17], [20]. A TMH is initially assigned when λ intersects the propensity profile at two consecutive points. For example, given λ = 0.4, the N-terminal residue of a TMH is residue n0 if E_n0−1_<λ and E_n0_>λ. Moving along the sequence, the next encounter of E_c0_>λ and E_c0+1_<λ defines the C-terminal residue of the TMH to be residue c0. Hence, the initial assignment of TMH is from residues n0 to c0. The value of λ was then increased by increment of 0.05 until λ intersects the profile within the initial TMH at four points. In this case, the original TMH was split into two TMH segments. The first TMH is from residues n0 to c1, where E_c1_>λ and E_c1+1_<λ, and the second TMH is from residues n1 to c0, where E_n1−1_<λ and E_n1_>λ. A TMH shorter than 5 residues was not segmented out and remained as a part of the original TMH. Figure 3 shows an example of dynamic threshold assignment of TMHs in the protein lactose permease of *Escherichia coli* (PDB code: 1PV7) [26]. Note that the short loops between the 3^rd^ and 4^th^ TMHs, and between the 9^th^ and 10^th^ TMHs were successfully detected using this method.

All algorithms used in MemBrain were implemented in the C programming language and executed in the Linux operating system.

## Supporting Information

Table S1 (0.02 MB DOC)

Table S2 (0.02 MB DOC)

Table S3 (0.05 MB DOC)

Data S1 (0.10 MB DOC)
