# Supplementary material for: MemBrain: Improving the Accuracy of Predicting Transmembrane Helices
Source: PLoS One. 2008 Jun 11;3(6):e2399. doi: 10.1371/journal.pone.0002399 (PMC2396505; doi:10.1371/journal.pone.0002399)
Supplement: Table S1 — (0.02 MB DOC) [file pone.0002399.s001.doc]

# Supplementary Table S1 for:

Hongbin Shen and James J. Chou, “MemBrain: Improving the Accuracy of Predicting Transmembrane Helices”

**Table S1**. The Swiss-Prot accession codes of the 50 proteins in the training dataset ^a^

P00396 P00404 P00415 P00423 P00430 P00844

P02699 P02724 P02945 P03617 P03621 P03805

P03806 P03989 P04038 P06008 P06009 P07470

P07471 P10175 P13183 P26789 P26790 Q54397

P07371 O53898 P77921 P00804 P02786 P02916

P08187 P08188 P08716 P15877 P17448 P18783

P19568 P21865 P22306 P22519 P23462 P23889

P24282 P25525 P25737 P37310 Q53068 P36574

P09130 P08336

^a^ The dataset is from ftp://ftp.ebi.ac.uk/pub/databases/testsets/transmembrane, in which the proteins labeled with grade ‘A’ or ‘B’ were used.
