# Supplementary material for: MemBrain: Improving the Accuracy of Predicting Transmembrane Helices
Source: PLoS One. 2008 Jun 11;3(6):e2399. doi: 10.1371/journal.pone.0002399 (PMC2396505; doi:10.1371/journal.pone.0002399)
Supplement: Table S2 — (0.02 MB DOC) [file pone.0002399.s002.doc]

# Supplementary Table S2 for:

Hongbin Shen and James J. Chou, “MemBrain: Improving the Accuracy of Predicting Transmembrane Helices”

**Table S2**. The PDB accession codes of the 70 proteins in the testing dataset

1AP9_A 1AR1_A 1AT9_A 1BCC_C 1EHK_A 1EYS_L

1EYS_M 1EYS_H 1FX8_A 1IH5_A 1IWG_A 1JGJ_A

1KQG_B 1KQG_C 1L7V_A 1LGH_A 1LGH_B 1NEK_C

1NEK_D 1NKZ_A 1OCC_D 1OCC_G 1OCC_J 1OCC_K

1OCC_L 1OCC_M 1OED_A 1OED_B 1OED_C 1OED_E

1OKC_A 1PRC_M 1PSS_L 1PSS_M 1PV7_A 1PW4_A

1Q90_D 1QHJ_A 1QLB_C 1RC2_B 1RHZ_A 1RWT_A

1SOR_A 1U7G_A 1UAZ_A 1VF5_C 1VF5_D 1VGO_A

1XIO_A 1XQF_A 1YCE_A 1ZCD_A 2A65_A 2AHZ_A

2B2J_A 2B5F_A 2BBJ_A 2BL2_A 2BRD_A 2H8A_A

2HI7_B 2IRV_A 2IUB_A 2J7A_C 2JO1_A 2NQ2_A

2ONK_C 2PNO_A 2Q7M_A 2QTS_A
