# Supplementary material for: MemBrain: Improving the Accuracy of Predicting Transmembrane Helices
Source: PLoS One. 2008 Jun 11;3(6):e2399. doi: 10.1371/journal.pone.0002399 (PMC2396505; doi:10.1371/journal.pone.0002399)
Supplement: Table S3 — (0.05 MB DOC) [file pone.0002399.s003.doc]

# Supplementary Table S3 for:

Hongbin Shen and James J. Chou, “MemBrain: Improving the Accuracy of Predicting Transmembrane Helices”

**Table S3**. Performance comparison of various TMH predictors for TMHs longer than 15 residues ^a^

| Predictor | V_TMH_ | N-score | C-score | *RMSD* |
| --- | --- | --- | --- | --- |
| THUMBU[16] ^b^ | 88.6% | 6.98±4.91 | 6.62±4.88 | 0.56±0.20 |
| SOSUI[11] ^c^ | 92.7% | 4.94±4.26 | 4.96±4.34 | 0.43±0.22 |
| DAS-TMfilter[20] ^d^ | 93.3% | 6.6±5.17 | 5.5±5.30 | 0.56±0.18 |
| TOP-PRED[1] ^e^ | 95.8% | 4.5±3.91 | 4.5±3.99 | 0.44±0.16 |
| TMHMM[6] ^f^ | 94.1% | 4.4±3.78 | 4.4±3.90 | 0.42±0.16 |
| Phobius[7] ^g^ | 95.0% | 4.5±3.91 | 4.3±4.10 | 0.44±0.19 |
| **MemBrain ^h^** | **99.2%** | **3.2±3.0** | **3.0±2.7** | **0.36±0.14** |

^a^ The testing dataset consists of 358 TMH segments from 70 proteins, where all TMHs are longer than 15 residues.

^b^ <http://sparks.informatics.iupui.edu/Softwares-Services_files/thumbup.htm> [16].

^c^ <http://bp.nuap.nagoya-u.ac.jp/sosui/> [11].

^d^ <http://mendel.imp.ac.at/sat/DAS/DAS.html> [20].

^e^ <http://bioweb.pasteur.fr/seqanal/interfaces/toppred.html> [1].

^f^ <http://www.cbs.dtu.dk/services/TMHMM/> [6].

^g^ <http://phobius.cgb.ki.se/> [7].

^h^ [http://chou.med.harvard.edu/bioinf/MemBrain/](http://chou.med.harvard.edu/bioinf/membrain/).
