# Supplementary material for: MemBrain: Improving the Accuracy of Predicting Transmembrane Helices
Source: PLoS One. 2008 Jun 11;3(6):e2399. doi: 10.1371/journal.pone.0002399 (PMC2396505; doi:10.1371/journal.pone.0002399)
Supplement: Data S1 — (0.10 MB DOC) [file pone.0002399.s004.doc]

# Supplementary Data S1 for:

Hongbin Shen and James J. Chou, “MemBrain: Improving the Accuracy of Predicting Transmembrane Helices”

Listed below are details of observed and predicted TMHs for the 70 membrane proteins in the testing dataset.

PDB code: 1AP9_A

Observed 10-30; 39-62; 77-101; 105-127; 134-157; 169-191; 202-224

MemBrain 10-29; 43-63; 78-97; 107-127; 134-154; 172-191; 203-224

THUMBU 16-27; 49-65; 85-106; 126-155; 179-194; 202-217

SOSUI 11-33; 46-68; 78-100; 107-129; 137-159; 177-199; 204-225

DAS-TMfilter 14-28; 47-67; 88-101; 110-123; 139-156; 178-190; 203-223

TOP-PRED 9-29; 44-64; 83-103; 106-126; 138-158; 176-196; 204-224

TMHMM 2.0 10-29; 42-64; 79-101; 108-130; 135-157; 177-199

Phobius 12-29; 41-63; 83-101; 108-129; 135-156; 177-199; 205-224

PDB code: 1AR1_A

Observed 27-59; 84-121; 130-151; 178-206; 218-251; 263-298; 304-322; 334-362; 370-395; 404-430; 441-468; 483-513

MemBrain 28-55; 86-118; 129-151; 177-205; 217-248; 264-294; 305-324; 334-361; 370-392; 406-435; 442-467; 482-512

THUMBU 36-51; 89-113; 133-146; 185-200; 216-244; 272-290; 308-327; 346-356; 371-390; 409-429; 449-459; 491-511

SOSUI 32-53; 90-112; 133-155; 181-203; 221-243; 276-298; 304-326; 339-361; 374-396; 408-430; 441-463; 492-514

DAS-TMfilter 32-52; 90-112; 137-150; 182-204; 215-246; 272-294; 303-325; 341-357; 373-392; 409-430; 450-460; 492-513

TOP-PRED 31-51; 93-113; 134-154; 157-177; 181-201; 217-237; 278-298; 305-325; 337-357; 372-392; 413-433; 446-466; 493-513

TMHMM 2.0 31-53; 90-112; 132-154; 183-205; 225-247; 272-294; 301-323; 338-360; 373-395; 410-432; 445-467; 491-513

Phobius 31-53; 91-115; 135-154; 181-207; 219-246; 266-290; 302-326; 338-361; 373-392; 412-432; 444-465; 491-512

PDB code: 1AT9_A

Observed 9-31; 37-62; 80-100; 106-126; 131-155; 165-191; 201-224

MemBrain 10-30; 43-63; 78-97; 107-127; 134-154; 172-191; 203-224

THUMBU 16-27; 49-65; 85-106; 126-155; 179-194; 202-217

SOSUI 10-32; 45-67; 77-99; 106-128; 136-158; 176-198; 203-224

DAS-TMfilter 14-28; 46-67; 88-101; 109-124; 138-156; 178-190; 203-223

TOP-PRED 8-28; 43-63; 82-102; 105-125; 137-157; 175-195; 203-223

TMHMM 2.0 10-29; 42-64; 79-101; 108-130; 135-157; 177-199

Phobius 12-29; 41-63; 83-101; 108-129; 135-156; 177-199; 205-224

PDB code: 1BCC_C

Observed 33-54; 76-106; 112-135; 172-204; 221-245; 289-309; 321-340; 346-379

MemBrain 33-54; 88-102; 114-134; 142-152; 179-200; 228-248; 291-307; 321-338; 351-372

THUMBU 33-51; 83-102; 121-139; 147-160; 180-197; 233-244; 293-303; 324-337; 353-366

SOSUI 33-55; 82-104; 114-136; 140-162; 179-201; 230-252; 280-302; 321-343; 349-371

DAS-TMfilter 36-55; 89-101; 117-133; 179-199; 231-247; 291-308; 324-339; 354-370

TOP-PRED 34-54; 82-102; 116-136; 150-170; 179-199; 230-250; 289-309; 321-341; 353-373

TMHMM 2.0 34-56; 83-105; 117-139; 144-166; 179-201; 230-252; 289-308; 323-341; 348-370

Phobius 31-53; 88-108; 114-134; 141-159; 179-201; 230-251; 289-309; 321-341; 347-367

PDB code: 1EHK_A

Observed 17-48; 65-97; 103-125; 143-173; 180-212; 220-254; 262-283; 292-326; 344-366; 380-409; 415-444; 461-493; 527-550

MemBrain 19-48; 66-96; 103-127; 142-169; 177-210; 221-252; 262-285; 292-325; 329-337; 344-365; 379-408; 419-444; 461-492; 529-545

THUMBU 24-40; 74-88; 105-128; 147-163; 182-216; 228-247; 265-279; 301-315; 352-360; 392-401; 421-445; 467-489; 526-547

SOSUI 21-43; 71-93; 109-131; 145-167; 189-211; 233-255; 262-284; 299-321; 341-363; 385-407; 421-443; 467-489; 527-549

DAS-TMfilter 21-39; 72-88; 108-126; 145-164; 181-216; 234-249; 265-279; 298-316; 347-359; 389-403; 422-441; 466-493; 527-547

TOP-PRED 21-41; 74-94; 103-123; 144-164; 182-202; 232-252; 264-284; 299-319; 345-365; 387-407; 420-440; 473-493; 527-547

TMHMM 2.0 21-43; 70-92; 105-127; 142-164; 187-209; 229-251; 264-286; 301-323; 343-365; 385-407; 420-442; 471-493; 527-549

Phobius 21-44; 64-92; 104-125; 145-164; 184-207; 227-251; 263-281; 301-324; 345-365; 385-408; 420-443; 463-489; 527-551

PDB code: 1EYS_L

Observed 32-56; 91-118; 123-141; 178-206; 233-258

MemBrain 30-56; 90-118; 122-146; 177-202; 233-260

THUMBU 22-53; 96-108; 127-151; 177-198; 231-259

SOSUI 12-34; 36-58; 93-115; 124-146; 235-257

DAS-TMfilter 24-54; 97-105; 123-148; 183-200; 232-259

TOP-PRED 31-51; 91-111; 122-142; 179-199; 240-260

TMHMM 2.0 29-51; 91-110; 123-145; 183-205; 240-262

Phobius 21-51; 92-110; 122-142; 182-205; 240-263

PDB code: 1EYS_M

Observed 53-77; 111-138; 143-161; 198-225; 262-285

MemBrain 52-76; 110-138; 142-166; 197-223; 266-288

THUMBU 53-79; 114-128; 145-167; 199-222; 264-292

SOSUI 51-73; 110-132; 145-167; 202-224; 268-290

DAS-TMfilter 50-76; 113-129; 147-165; 202-222; 271-289

TOP-PRED 52-72; 110-130; 143-163; 200-220; 268-288

TMHMM 2.0 50-72; 111-133; 145-167; 203-225; 267-289

Phobius 50-76; 113-133; 145-166; 200-223; 267-290

PDB code: 1EYS_H

Observed 10-34

MemBrain 11-29

THUMBU 10-26

SOSUI 9-30

DAS-TMfilter 16-27

TOP-PRED 11-31; 83-103

TMHMM 2.0 13-30

Phobius 12-30

PDB code: 1FX8_A

Observed 6-35; 40-64; 68-79; 85-109; 144-168; 178-199; 203-217; 233-255

MemBrain 10-31; 40-62; 68-78; 84-108; 146-165; 178-198; 211-219; 232-254

THUMBU 15-33; 43-74; 94-110; 149-164; 180-195; 236-247

SOSUI 15-37; 43-65; 86-108; 145-167; 178-200; 230-251

DAS-TMfilter 15-32; 46-61; 70-79; 94-110; 150-166; 178-194; 233-249

TOP-PRED 12-32; 44-64; 86-106; 148-168; 177-197; 230-250

TMHMM 2.0 12-34; 44-66; 87-109; 143-165; 178-200; 229-251

Phobius 12-34; 46-79; 86-108; 145-166; 178-202; 230-251

PDB code: 1IH5_A

Observed 10-34; 47-64; 77-88; 96-112; 140-159; 169-184; 189-202; 210-229

MemBrain 12-35; 48-69; 76-87; 91-117; 138-156; 166-187; 209-230

THUMBU 16-32; 82-112; 173-183; 213-227

SOSUI 13-35; 87-109; 134-155; 167-189; 205-227

DAS-TMfilter 15-33; 78-115; 78-115; 143-153; 170-184; 214-231

TOP-PRED 15-35; 52-72; 75-95; 101-121; 138-158; 164-184; 207-227

TMHMM 2.0 13-35; 65-87; 94-116; 136-155; 167-189; 209-231

Phobius 9-33; 53-73; 94-119; 139-157; 164-184; 210-231

PDB code: 1IWG_A

Observed 9-28; 335-360; 365-385; 392-413; 440-460; 472-495; 541-560; 863-888; 894-916; 927-949; 976-996; 1011-1029

MemBrain 9-28; 341-361; 364-387; 391-410; 438-464; 466-495; 538-560; 873-893; 895-922; 923-943; 971-992; 1001-1029

THUMBU 10-27; 341-359; 390-408; 443-459; 471-492; 538-557; 877-914; 974-991; 1010-1026

SOSUI 9-31; 335-357; 367-389; 396-418; 441-463; 475-497; 541-563; 872-894; 897-919; 973-995; 1007-1029

DAS-TMfilter 10-29; 342-362; 365-410; 365-410; 442-459; 472-494; 540-558; 876-917; 876-917; 975-992; 1010-1029

TOP-PRED 9-29; 32-52; 131-151; 342-362; 370-390; 441-461; 470-490; 541-561; 660-680; 872-892; 898-918; 926-946; 974-994; 1009-1029

TMHMM 2.0 10-32; 340-359; 369-391; 438-460; 470-492; 542-564; 873-892; 899-921; 926-948; 975-997; 1007-1029

Phobius 12-32; 340-359; 366-390; 396-416; 437-458; 470-493; 541-557; 875-892; 899-919; 925-946; 975-995; 1007-1029

PDB code: 1JGJ_A

Observed 1-27; 33-56; 70-92; 94-118; 121-150; 153-181; 189-216

MemBrain 6-25; 36-60; 70-88; 97-119; 122-144; 161-181; 190-211

THUMBU 10-22; 43-62; 75-95; 104-113; 122-141; 164-183; 185-208

SOSUI 4-26; 39-61; 74-95; 98-120; 126-148; 156-178; 189-211

DAS-TMfilter 8-20; 41-58; 77-92; 101-114; 128-142; 167-182; 184-206

TOP-PRED 4-24; 38-58; 74-94; 98-118; 124-144; 165-185; 193-213

TMHMM 2.0 4-26; 39-61; 76-93; 98-120; 124-146; 167-189; 194-213

Phobius 6-25; 37-61; 73-91; 98-118; 124-142; 163-182; 194-213

PDB code: 1KQG_B

Observed 256-280

MemBrain 257-275

THUMBU 264-274

SOSUI 258-280

DAS-TMfilter 262-275

TOP-PRED 130-150; 259-279

TMHMM 2.0 260-279

Phobius 260-279

PDB code: 1KQG_C

Observed 11-37; 49-76; 110-135; 144-176

MemBrain 17-40; 57-75; 115-136; 146-177

THUMBU 15-38; 56-74; 118-141; 151-176

SOSUI 18-40; 55-77; 115-137; 150-172

DAS-TMfilter 18-39; 56-76; 117-137; 149-175

TOP-PRED 20-40; 55-75; 115-135; 156-176

TMHMM 2.0 21-40; 55-77; 117-139; 154-176

Phobius 21-43; 55-76; 117-138; 150-174

PDB code: 1L7V_A

Observed 2-32; 47-81; 93-107; 114-138; 142-166; 191-206; 229-249; 258-267; 272-296; 305-324

MemBrain 14-34; 55-77; 94-111; 114-136; 144-164; 191-208; 231-256; 274-295; 300-320

THUMBU 18-32; 57-74; 118-134; 147-164; 192-207; 232-267; 280-305; 307-317

SOSUI 13-34; 52-74; 102-124; 141-163; 184-200; 228-250; 265-287; 292-314

DAS-TMfilter 16-33; 59-74; 97-107; 117-135; 145-163; 193-204; 242-266; 279-319; 279-319

TOP-PRED 12-32; 58-78; 85-105; 110-130; 142-162; 181-201; 230-250; 263-283; 292-312

TMHMM 2.0 13-35; 55-77; 89-111; 115-137; 144-166; 239-261; 274-296; 300-322

Phobius 58-77; 89-109; 115-133; 145-168; 188-206; 213-231; 243-263; 275-297; 303-322

PDB code: 1LGH_A

Observed 15-39

MemBrain 15-38

THUMBU 15-42

SOSUI 9-31; 34-56

DAS-TMfilter 16-43

TOP-PRED 16-36

TMHMM 2.0 21-43

Phobius 20-48

PDB code: 1LGH_B

Observed 10-41

MemBrain 20-39

THUMBU 27-36

SOSUI 20-42

DAS-TMfilter 26-41

TOP-PRED 22-42

TMHMM 2.0 22-41

Phobius 20-41

PDB code: 1NEK_C

Observed 21-54; 67-97; 102-129

MemBrain 27-59; 63-99; 109-123

THUMBU 26-49; 66-91

SOSUI 25-47; 65-87; 109-129

DAS-TMfilter 26-50; 68-88; 113-125

TOP-PRED 32-52; 62-82; 109-129

TMHMM 2.0 27-49; 64-86; 112-128

Phobius 20-48; 68-89; 110-128

PDB code: 1NEK_D

Observed 11-38; 54-84; 86-114

MemBrain 19-40; 49-78; 91-109

THUMBU 19-45; 51-80; 88-106

SOSUI 16-38; 51-73; 89-111

DAS-TMfilter 18-37; 54-74; 88-111

TOP-PRED 21-41; 59-79; 95-115

TMHMM 2.0 16-38; 48-70; 91-113

Phobius 17-39; 59-80; 92-113

PDB code: 1NKZ_A

Observed 11-36

MemBrain 13-35

THUMBU 15-40

SOSUI 11-33

DAS-TMfilter 13-38

TOP-PRED 12-32

TMHMM 2.0 13-35

Phobius 14-35

PDB code: 1OCC_D

Observed 77-103

MemBrain 77-101

THUMBU 85-98

SOSUI 79-101

DAS-TMfilter 84-97

TOP-PRED 79-99

TMHMM 2.0 81-98

Phobius 80-98

PDB code: 1OCC_G

Observed 13-37

MemBrain 14-36

THUMBU 21-32

SOSUI 16-37

DAS-TMfilter 21-30

TOP-PRED 18-38

TMHMM 2.0 15-37

Phobius 16-37

PDB code: 1OCC_J

Observed 26-54

MemBrain 26-52

THUMBU 38-48

SOSUI 33-54

DAS-TMfilter 35-48

TOP-PRED 34-54

TMHMM 2.0 30-52

Phobius 30-53

PDB code: 1OCC_K

Observed 9-35

MemBrain 10-34

THUMBU 21-34

SOSUI 14-36

DAS-TMfilter 18-34

TOP-PRED 14-34

TMHMM 2.0 13-35

Phobius 12-32

PDB code: 1OCC_L

Observed 18-44

MemBrain 18-41

THUMBU 26-36

SOSUI 20-42

DAS-TMfilter 24-35

TOP-PRED 20-40

TMHMM 2.0 20-42

Phobius 20-40

PDB code: 1OCC_M

Observed 12-35

MemBrain 12-34

THUMBU 23-32

SOSUI 15-37

DAS-TMfilter 19-34

TOP-PRED 15-35

TMHMM 2.0 15-37

Phobius 16-35

PDB code: 1OED_A

Observed 2-29; 32-61; 65-92; 194-227

MemBrain 6-24; 35-50; 61-87; 194-219

THUMBU 10-22; 39-56; 65-84; 194-216

SOSUI 3-25; 35-57; 64-86; 196-218

DAS-TMfilter 6-25; 35-57; 63-86; 194-217

TOP-PRED 7-27; 33-53; 67-87; 198-218

TMHMM 2.0 4-26; 35-57; 67-89; 199-221

Phobius 6-25; 37-55; 67-86; 199-221

PDB code: 1OED_B

Observed 2-29; 32-61; 67-91; 216-246

MemBrain 6-25; 35-50; 61-87; 217-241

THUMBU 10-22; 38-50; 63-84; 219-238

SOSUI 3-25; 32-54; 63-85; 218-240

DAS-TMfilter 6-26; 36-52; 60-87; 218-238

TOP-PRED 5-25; 32-52; 67-87; 222-242

TMHMM 2.0 4-26; 33-51; 66-88; 222-240

Phobius 6-26; 33-51; 63-88; 222-240

PDB code: 1OED_C

Observed 2-29; 32-61; 65-92; 228-260

MemBrain 6-24; 35-50; 61-87; 227-252

THUMBU 10-22; 65-84; 233-250

SOSUI 4-26; 32-54; 64-86; 231-253

DAS-TMfilter 8-24; 37-50; 61-86; 233-251

TOP-PRED 7-27; 32-52; 67-87; 232-252

TMHMM 2.0 5-27; 34-53; 68-90; 232-254

Phobius 6-25; 37-55; 67-88; 232-254

PDB code: 1OED_E

Observed 3-28; 32-62; 67-93; 227-260

MemBrain 6-25; 36-51; 62-88; 227-251

THUMBU 10-23; 66-87; 229-249

SOSUI 4-26; 35-57; 67-89; 229-251

DAS-TMfilter 7-26; 38-52; 64-89; 232-251

TOP-PRED 7-27; 33-53; 68-88; 232-252

TMHMM 2.0 5-27; 34-53; 68-90; 232-254

Phobius 6-24; 36-56; 68-91; 232-254

PDB code: 1OKC_A

Observed 8-27; 66-89; 108-136; 169-197; 206-230; 266-290

MemBrain 10-29; 70-89; 112-134; 173-193; 211-230; 270-290

THUMBU 214-222

SOSUI NONE

DAS-TMfilter NONE

TOP-PRED 9-29; 113-133; 207-227

TMHMM 2.0 111-133; 171-193; 208-230

Phobius 171-189; 209-230

PDB code: 1PRC_M

Observed 52-76; 111-137; 143-166; 198-223; 260-284

MemBrain 50-76; 110-137; 141-165; 196-222; 265-287

THUMBU 52-77; 109-127; 143-164; 200-221; 265-292

SOSUI 52-74; 108-130; 141-163; 202-224; 267-289

DAS-TMfilter 51-74; 114-125; 144-164; 204-222; 269-288

TOP-PRED 54-74; 109-129; 144-164; 199-219; 266-286

TMHMM 2.0 49-71; 110-132; 144-166; 203-225; 267-289

Phobius 49-75; 112-132; 144-165; 202-225; 266-289

PDB code: 1PSS_L

Observed 32-55; 83-111; 116-138; 171-198; 225-250

MemBrain 27-51; 78-106; 110-134; 165-192; 221-249

THUMBU 18-53; 85-95; 112-146; 172-187; 223-254

SOSUI 24-46; 78-100; 111-133; 170-192; 225-247

DAS-TMfilter 19-50; 85-93; 111-136; 171-191; 220-249

TOP-PRED 27-47; 79-99; 110-130; 168-188; 229-249

TMHMM 2.0 25-47; 79-101; 108-130; 171-193; 230-252

Phobius 21-47; 80-98; 110-130; 170-194; 228-252

PDB code: 1PSS_M

Observed 54-78; 109-139; 147-168; 200-226; 262-286

MemBrain 41-71; 108-134; 138-162; 193-219; 262-284

THUMBU 41-80; 107-128; 142-163; 193-218; 260-286

SOSUI 47-69; 106-127; 141-163; 200-222; 267-289

DAS-TMfilter 45-69; 109-122; 144-162; 198-219; 265-284

TOP-PRED 25-45; 50-70; 106-126; 140-160; 196-216; 263-283

TMHMM 2.0 49-71; 107-129; 141-163; 199-221; 263-285

Phobius 45-71; 108-130; 142-162; 199-221; 263-286

PDB code: 1PV7_A

Observed 6-39; 41-59; 74-101; 103-137; 139-165; 165-186; 220-248; 253-277; 287-308; 311-341; 357-377; 377-400

MemBrain 8-33; 45-64; 74-100; 102-125; 141-163; 167-187; 211-241; 256-281; 290-307; 312-335; 349-372; 379-399

THUMBU 10-32; 49-59; 77-95; 103-121; 149-187; 268-279; 320-330; 345-365; 379-397

SOSUI 11-33; 44-66; 75-97; 106-128; 144-166; 174-196; 215-237; 260-282; 288-310; 313-335; 346-368; 379-401

DAS-TMfilter 11-30; 48-64; 76-116; 76-116; 153-162; 171-187; 224-232; 294-302; 320-332; 348-367; 381-400

TOP-PRED 7-27; 45-65; 78-98; 102-122; 167-187; 219-239; 263-283; 291-311; 315-335; 349-369; 382-402

TMHMM 2.0 13-35; 45-67; 76-98; 103-125; 145-164; 168-187; 222-239; 261-283; 304-326; 346-368; 380-402

Phobius 12-34; 46-66; 75-95; 101-122; 143-162; 168-187; 222-239; 259-279; 291-310; 316-337; 349-369; 381-401

PDB code: 1PW4_A

Observed 19-58; 63-89; 93-113; 120-148; 152-181; 189-208; 252-283; 287-317; 321-342; 346-375; 379-410; 414-449

MemBrain 26-53; 62-84; 93-114; 116-144; 156-182; 185-206; 253-279; 286-313; 320-344; 346-371; 381-408; 412-434

THUMBU 30-46; 98-133; 165-207; 251-271; 327-337; 345-368; 388-406; 415-433

SOSUI 26-43; 102-124; 160-181; 187-208; 251-273; 290-312; 320-342; 350-372; 384-406; 414-435

DAS-TMfilter 29-42; 68-77; 94-132; 94-132; 170-181; 189-206; 252-271; 324-338; 349-371; 387-406; 415-436

TOP-PRED 25-45; 66-86; 94-114; 163-183; 187-207; 252-272; 292-312; 321-341; 350-370; 384-404; 415-435

TMHMM 2.0 27-44; 64-86; 93-115; 119-141; 154-176; 186-205; 253-272; 292-311; 318-340; 350-372; 385-407; 417-436

Phobius 25-43; 63-81; 93-112; 118-137; 158-182; 188-207; 253-271; 291-310; 322-341; 347-372; 384-404; 416-436

PDB code: 1Q90_D

Observed 38-58; 93-109; 126-147

MemBrain 34-57; 95-113; 128-146

THUMBU 39-54; 88-109; 132-150

SOSUI 36-58; 89-111; 131-153

DAS-TMfilter 38-55; 94-110; 129-147

TOP-PRED 37-57; 94-114; 127-147

TMHMM 2.0 36-58; 95-117; 129-151

Phobius 36-57; 95-114; 126-145

PDB code: 1QHJ_A

Observed 6-32; 37-58; 80-100; 105-127; 131-160; 165-191; 201-224

MemBrain 10-29; 43-63; 78-97; 107-127; 134-154; 172-191; 203-224

THUMBU 16-27; 49-65; 85-106; 126-155; 179-194; 202-217

SOSUI 11-33; 46-68; 78-100; 107-129; 137-159; 177-199; 204-225

DAS-TMfilter 14-28; 47-67; 88-101; 109-124; 138-156; 178-190; 203-223

TOP-PRED 9-29; 44-64; 83-103; 106-126; 138-158; 176-196; 204-224

TMHMM 2.0 10-29; 42-64; 79-101; 108-130; 135-157; 177-199

Phobius 12-29; 41-63; 83-101; 108-129; 135-156; 177-199; 205-224

PDB code: 1QLB_C

Observed 22-52; 77-100; 121-149; 169-194; 202-237

MemBrain 32-55; 78-95; 128-145; 166-181; 214-229

THUMBU 33-54; 76-96; 129-146; 166-185; 218-228

SOSUI 33-55; 77-98; 129-151; 171-193; 213-232

DAS-TMfilter 33-57; 76-97; 131-147; 167-186; 214-231

TOP-PRED 32-52; 76-96; 123-143; 163-183; 212-232

TMHMM 2.0 31-53; 76-98; 125-147; 167-189; 215-232

Phobius 28-53; 77-98; 125-147; 167-191; 212-232

PDB code: 1RC2_B

Observed 3-26; 34-54; 63-73; 79-104; 130-154; 161-182; 186-198; 206-225

MemBrain 6-24; 35-57; 64-73; 79-104; 132-151; 161-182; 204-224

THUMBU 13-25; 28-54; 87-103; 134-145; 164-177; 199-219

SOSUI 5-27; 35-57; 81-103; 131-153; 161-183; 205-227

DAS-TMfilter 13-26; 32-55; 85-103; 132-150; 162-179; 206-221

TOP-PRED 9-29; 35-55; 85-105; 130-150; 156-176; 203-223

TMHMM 2.0 7-29; 33-55; 81-103; 127-149; 156-178; 205-227

Phobius 36-60; 81-103; 129-149; 161-180; 206-226

PDB code: 1RHZ_A

Observed 22-41; 59-89; 105-129; 136-161; 168-188; 206-229; 255-276; 312-333; 375-396; 400-415

MemBrain 26-44; 56-66; 71-90; 111-132; 136-159; 167-192; 205-227; 253-282; 313-336; 375-396; 398-413

THUMBU 34-50; 74-92; 114-132; 140-158; 173-181; 209-229; 256-280; 312-333; 381-407

SOSUI 31-53; 71-93; 110-132; 138-160; 167-189; 208-230; 258-280; 313-335; 384-406

DAS-TMfilter 31-44; 69-90; 115-157; 115-157; 170-179; 211-226; 255-271; 314-333; 381-410

TOP-PRED 27-47; 67-87; 125-145; 168-188; 209-229; 257-277; 292-312; 315-335; 374-394

TMHMM 2.0 33-55; 70-92; 113-135; 139-157; 164-186; 209-231; 252-274; 313-335; 383-405

Phobius 29-47; 67-93; 113-132; 138-157; 169-189; 209-226; 247-269; 316-335; 373-391; 397-415

PDB code: 1RWT_A

Observed 55-87; 124-144; 170-201

MemBrain 56-87; 122-141; 179-198

THUMBU 124-137

SOSUI 118-140

DAS-TMfilter NONE

TOP-PRED 66-86; 120-140; 184-204

TMHMM 2.0 NONE

Phobius NONE

PDB code: 1SOR_A

Observed 5-32; 40-63; 68-78; 82-108; 126-150; 159-180; 184-194; 200-222

MemBrain 8-29; 35-57; 64-75; 77-106; 125-144; 154-175; 197-219

THUMBU 10-29; 37-55; 74-96; 129-140; 160-171; 202-214

SOSUI 5-27; 35-57; 75-97; 122-143; 154-174; 193-215

DAS-TMfilter 11-26; 37-54; 78-101; 128-142; 157-170; 205-218

TOP-PRED 8-28; 36-56; 82-102; 126-146; 153-173; 197-217

TMHMM 2.0 5-27; 37-59; 79-101; 121-143; 156-178; 198-220

Phobius 9-31; 37-58; 79-103; 123-144; 156-175; 198-220

PDB code: 1U7G_A

Observed 6-34; 37-69; 96-120; 124-149; 163-182; 194-221; 224-254; 257-273; 280-307; 310-333; 347-381

MemBrain 12-34; 45-68; 97-118; 125-149; 165-181; 199-216; 225-249; 260-275; 277-301; 317-337; 347-379

THUMBU 17-29; 48-66; 99-117; 129-147; 165-180; 204-215; 231-254; 266-299; 319-335; 355-374

SOSUI 11-33; 41-63; 111-133; 155-176; 187-209; 217-239; 245-267; 270-292; 307-329; 347-369

DAS-TMfilter 16-32; 48-64; 107-119; 124-144; 169-180; 230-247; 262-271; 277-297; 321-333; 359-373

TOP-PRED 11-31; 41-61; 94-114; 119-139; 154-174; 190-210; 219-239; 246-266; 273-293; 310-330; 345-365

TMHMM 2.0 10-32; 41-63; 98-120; 127-149; 164-183; 196-215; 225-247; 254-276; 280-302; 315-337; 352-374

Phobius 12-32; 44-64; 98-120; 127-149; 161-183; 195-214; 226-247; 254-272; 278-302; 314-338; 358-377

PDB code: 1UAZ_A

Observed 15-38; 44-69; 86-106; 110-133; 136-160; 170-198; 206-231

MemBrain 17-36; 49-71; 85-104; 112-134; 139-161; 178-198; 209-230

THUMBU 22-34; 55-70; 90-103; 118-132; 141-159; 179-197; 205-223

SOSUI 17-39; 50-72; 89-111; 113-135; 142-164; 177-199; 210-231

DAS-TMfilter 19-35; 54-70; 94-107; 117-132; 144-161; 179-197; 205-229

TOP-PRED 16-36; 53-73; 89-109; 114-134; 142-162; 179-199; 210-230

TMHMM 2.0 15-35; 48-70; 85-107; 114-133; 143-162; 175-197; 207-229

Phobius 17-35; 47-69; 89-107; 114-135; 141-159; 180-198; 204-230

PDB code: 1VF5_C

Observed 251-280

MemBrain 255-271

THUMBU 256-270

SOSUI NONE

DAS-TMfilter 260-270

TOP-PRED 255-275

TMHMM 2.0 257-274

Phobius 257-274

PDB code: 1VF5_D

Observed 18-43

MemBrain 19-41

THUMBU 25-36

SOSUI NONE

DAS-TMfilter 24-37

TOP-PRED 18-38; 101-121

TMHMM 2.0 21-43

Phobius 21-39

PDB code: 1VGO_A

Observed 13-36; 40-66; 85-107; 109-132; 135-159; 169-196; 205-230

MemBrain 15-34; 47-68; 84-102; 111-133; 138-159; 177-197; 207-229

THUMBU 20-32; 52-72; 88-102; 116-126; 143-159; 181-204

SOSUI 12-34; 55-77; 88-110; 112-134; 141-163; 175-197; 209-230

DAS-TMfilter 17-32; 51-69; 92-106; 114-130; 143-160; 179-196; 206-228

TOP-PRED 13-33; 51-71; 88-108; 112-132; 140-160; 178-198; 209-229

TMHMM 2.0 15-33; 46-68; 83-105; 110-132; 142-161; 174-196; 206-228

Phobius 15-33; 45-68; 88-106; 113-134; 140-158; 179-197; 203-229

PDB code: 1XIO_A

Observed 2-27; 33-57; 69-92; 98-122; 124-148; 158-186; 194-224

MemBrain 6-25; 36-58; 69-89; 99-122; 127-149; 166-186; 196-219

THUMBU 40-50; 75-87; 130-146; 168-186; 196-212

SOSUI 4-26; 33-55; 74-95; 101-123; 127-149; 169-191

DAS-TMfilter 39-50; 78-89; 103-116; 131-146; 169-185; 199-212

TOP-PRED 5-25; 37-57; 75-95; 99-119; 129-149; 168-188; 196-216

TMHMM 2.0 5-24; 37-56; 76-95; 100-119; 129-147; 168-187; 197-216

Phobius 6-25; 37-56; 76-92; 99-117; 129-147; 168-193; 199-218

PDB code: 1XQF_A

Observed 6-36; 43-69; 97-120; 124-149; 167-181; 197-218; 224-254; 257-273; 280-301; 313-333; 347-381

MemBrain 9-33; 44-66; 96-118; 126-148; 164-181; 198-216; 225-249; 260-274; 277-301; 317-334; 349-380

THUMBU 12-30; 47-67; 97-115; 137-155; 165-180; 204-215; 231-245; 266-299; 319-337; 354-374

SOSUI 12-34; 44-66; 118-140; 162-183; 194-216; 225-247; 278-300; 318-340; 357-379

DAS-TMfilter 13-33; 45-66; 107-120; 123-148; 169-181; 202-212; 229-246; 276-299; 320-335; 358-374

TOP-PRED 11-31; 44-64; 100-120; 125-145; 161-181; 197-217; 227-247; 256-276; 282-302; 319-339; 356-376

TMHMM 2.0 10-32; 45-67; 98-120; 127-149; 164-183; 196-215; 225-247; 259-281; 285-302; 315-337; 352-374

Phobius 12-32; 44-64; 98-120; 127-149; 161-183; 195-214; 226-246; 258-275; 281-302; 314-334; 354-374

PDB code: 1YCE_A

Observed 2-46; 49-81

MemBrain 8-37; 55-83

THUMBU 65-80

SOSUI 5-27; 67-89

DAS-TMfilter 69-82

TOP-PRED 8-28; 69-89

TMHMM 2.0 9-31; 66-88

Phobius 69-88

PDB code: 1ZCD_A

Observed 12-30; 59-85; 95-116; 121-143; 150-175; 182-200; 205-218; 223-236; 247-271; 290-311; 327-350; 357-382

MemBrain 15-28; 63-77; 98-115; 131-144; 150-178; 183-199; 204-219; 225-235; 257-269; 285-312; 328-348; 363-379

THUMBU 12-30; 62-72; 96-114; 133-151; 173-191; 214-232; 258-272; 287-307; 330-346; 366-375

SOSUI 11-32; 58-79; 93-115; 126-148; 153-175; 181-202; 212-234; 253-274; 291-313; 327-349; 357-379

DAS-TMfilter 14-28; 65-77; 98-114; 134-176; 134-176; 183-199; 207-220; 226-236; 258-270; 285-312; 330-349; 365-374

TOP-PRED 12-32; 58-78; 95-115; 126-146; 154-174; 179-199; 220-240; 254-274; 282-302; 328-348; 360-380

TMHMM 2.0 7-29; 60-77; 94-116; 126-145; 152-174; 179-201; 206-237; 257-279; 291-313; 328-350; 357-379

Phobius 12-39; 59-79; 91-114; 126-145; 154-175; 181-200; 207-238; 258-276; 288-312; 324-351; 363-380

PDB code: 2A65_A

Observed 10-38; 40-71; 87-125; 165-184; 190-214; 240-267; 275-307; 336-370; 374-396; 398-425; 446-478; 482-514

MemBrain 14-27; 42-63; 77-123; 163-188; 196-218; 249-265; 277-304; 334-366; 378-400; 405-428; 445-471; 485-502

THUMBU 16-24; 42-62; 87-119; 167-182; 199-215; 249-261; 297-318; 333-359; 382-394; 411-425; 453-468; 484-501

SOSUI 6-28; 41-63; 88-110; 112-133; 164-186; 196-218; 243-265; 292-314; 340-362; 377-399; 407-429; 453-475; 483-504

DAS-TMfilter 43-60; 92-124; 165-184; 198-216; 247-264; 297-309; 337-358; 380-395; 409-427; 452-468; 485-503

TOP-PRED 9-29; 41-61; 91-111; 164-184; 194-214; 247-267; 291-311; 335-355; 377-397; 409-429; 449-469; 485-505

TMHMM 2.0 7-29; 39-61; 89-111; 165-187; 194-216; 243-265; 293-315; 335-357; 378-395; 405-427; 447-469; 484-503

Phobius 12-29; 41-61; 92-111; 166-185; 197-215; 243-268; 289-314; 342-367; 379-399; 405-427; 447-469; 481-503

PDB code: 2AHZ_A

Observed 21-45; 49-62; 73-103

MemBrain 21-44; 51-74; 78-103

THUMBU 31-39; 81-100

SOSUI 1-20; 24-46; 81-103

DAS-TMfilter 27-40; 79-96

TOP-PRED 24-44; 50-70; 76-96

TMHMM 2.0 26-48; 78-100

Phobius 26-45; 78-100

PDB code: 2B2J_A

Observed 2-29; 35-64; 84-105; 113-138; 156-172; 186-208; 213-241; 245-261; 268-293; 300-320; 334-367

MemBrain 7-29; 38-63; 86-107; 114-137; 146-170; 187-206; 213-239; 248-263; 266-289; 305-320; 338-366

THUMBU 10-25; 42-63; 90-103; 116-139; 154-164; 189-206; 223-234; 268-284; 344-363

SOSUI 7-29; 38-60; 62-84; 88-110; 113-135; 148-170; 182-203; 215-237; 258-280; 306-328; 343-365

DAS-TMfilter 9-26; 39-61; 93-106; 116-133; 154-167; 189-201; 227-237; 251-260; 270-286; 342-363

TOP-PRED 6-26; 39-59; 64-84; 87-107; 114-134; 152-172; 186-206; 220-240; 245-265; 269-289; 301-321; 340-360

TMHMM 2.0 6-28; 35-57; 87-109; 116-138; 153-172; 185-204; 219-238; 245-267; 272-289; 301-323; 338-360

Phobius 6-28; 40-59; 87-105; 117-138; 150-172; 184-204; 216-240; 247-265; 271-287; 299-319; 339-360

PDB code: 2B5F_A

Observed 39-64; 73-92; 101-110; 115-139; 160-182; 198-218; 222-234; 243-260

MemBrain 40-60; 72-95; 101-111; 117-141; 163-182; 195-218; 241-263

THUMBU 40-56; 72-97; 111-133; 200-215; 246-260

SOSUI 40-62; 70-92; 114-136; 163-185; 192-214; 240-262

DAS-TMfilter 41-60; 70-95; 105-135; 167-180; 197-215; 248-257

TOP-PRED 43-63; 71-91; 119-139; 164-184; 194-214; 241-261

TMHMM 2.0 40-62; 72-94; 115-137; 162-181; 194-216; 242-264

Phobius 37-60; 72-95; 115-137; 196-216; 243-264

PDB code: 2BBJ_A

Observed 293-312; 326-345

MemBrain 295-313; 327-347

THUMBU 299-313; 332-342

SOSUI 295-317; 328-347

DAS-TMfilter 297-311; 332-345

TOP-PRED 296-316; 328-348

TMHMM 2.0 296-318; 328-348

Phobius 296-316; 328-348

PDB code: 2BL2_A

Observed 11-46; 51-79; 87-122; 127-155

MemBrain 13-40; 55-80; 89-114; 127-150

THUMBU 10-23; 63-85; 92-102; 136-147

SOSUI 7-29; 63-85; 94-116; 131-153

DAS-TMfilter 14-23; 67-80; 138-151

TOP-PRED 10-30; 62-82; 89-109; 136-156

TMHMM 2.0 13-35; 55-77; 89-111; 131-153

Phobius 55-78; 90-111; 131-154

PDB code: 2BRD_A

Observed 9-31; 38-62; 77-100; 105-127; 134-157; 166-191; 203-226

MemBrain 10-30; 43-63; 78-97; 107-127; 134-154; 172-191; 203-224

THUMBU 16-27; 49-65; 85-106; 126-155; 179-194; 202-217

SOSUI 10-32; 45-67; 77-99; 106-128; 136-158; 176-198; 203-224

DAS-TMfilter 14-28; 46-67; 88-102; 109-124; 138-156; 178-191; 203-223

TOP-PRED 8-28; 43-63; 82-102; 105-125; 137-157; 175-195; 203-223

TMHMM 2.0 10-29; 42-64; 79-101; 108-130; 135-157; 177-199

Phobius 12-29; 41-63; 83-101; 108-129; 135-156; 177-199; 205-224

PDB code: 2H8A_A

Observed 9-32; 62-95; 99-122; 128-147

MemBrain 13-33; 83-95; 101-117; 133-143

THUMBU 18-27; 106-117

SOSUI NONE

DAS-TMfilter 18-26; 85-93

TOP-PRED 11-31; 80-100; 128-148

TMHMM 2.0 10-32; 82-104; 130-148

Phobius 12-35; 82-110; 130-148

PDB code: 2HI7_B

Observed 14-36; 43-64; 71-96; 144-162

MemBrain 13-33; 39-64; 69-91; 140-164

THUMBU 18-60; 76-85; 142-160

SOSUI 12-34; 41-63; 144-165

DAS-TMfilter 16-38; 41-62; 72-80; 145-163

TOP-PRED 13-33; 48-68; 144-164

TMHMM 2.0 13-35; 45-64; 71-89; 145-162

Phobius 12-33; 45-64; 71-89; 145-166

PDB code: 2IRV_A

Observed 3-24; 56-79; 79-103; 109-127; 137-150; 161-178

MemBrain 6-21; 46-74; 79-103; 106-123; 132-156; 162-175

THUMBU 10-27; 51-70; 136-173

SOSUI 2-24; 47-69; 78-100; 102-123; 134-156; 159-180

DAS-TMfilter 8-22; 52-69; 83-94; 110-118; 136-153; 165-173

TOP-PRED 3-23; 51-71; 78-98; 102-122; 137-157

TMHMM 2.0 5-24; 44-66; 78-97; 101-123; 132-154; 159-181

Phobius 7-24; 44-69; 81-97; 103-121; 133-151; 157-176

PDB code: 2IUB_A

Observed 291-312; 327-349

MemBrain 304-322; 336-356

THUMBU 308-322; 341-351

SOSUI 304-326; 337-356

DAS-TMfilter 306-320; 341-354

TOP-PRED 305-325; 337-357

TMHMM 2.0 305-327; 337-357

Phobius 305-325; 337-357

PDB code: 2J7A_C

Observed 14-38

MemBrain 13-32

THUMBU 19-29

SOSUI 14-34

DAS-TMfilter 16-31

TOP-PRED 15-35

TMHMM 2.0 13-32

Phobius NONE

PDB code: 2JO1_A

Observed 18-38

MemBrain 19-36

THUMBU 18-36

SOSUI 16-37

DAS-TMfilter 16-38

TOP-PRED 17-37

TMHMM 2.0 15-37

Phobius 16-36

PDB code: 2NQ2_A

Observed 7-24; 57-87; 99-113; 119-138; 147-171; 199-213; 236-256; 265-274; 279-303; 311-329

MemBrain 8-29; 62-84; 100-118; 120-141; 148-170; 196-215; 238-262; 281-301; 307-327

THUMBU 13-26; 65-80; 103-136; 138-165; 197-215; 239-270; 286-296

SOSUI 7-29; 65-87; 93-115; 119-141; 149-171; 196-218; 239-261; 281-303; 308-329

DAS-TMfilter 10-27; 67-82; 107-139; 146-167; 198-215; 240-252; 287-295; 312-326

TOP-PRED 8-28; 66-86; 120-140; 146-166; 196-216; 240-260; 279-299; 308-328

TMHMM 2.0 10-32; 66-88; 108-139; 146-168; 198-220; 241-263; 309-328

Phobius 6-27; 66-87; 107-140; 147-171; 197-219; 240-267; 279-297; 309-328

PDB code: 2ONK_C

Observed 24-53; 67-101; 106-134; 150-177; 199-228; 252-273

MemBrain 23-58; 70-100; 110-136; 142-172; 203-236; 249-273

THUMBU 25-53; 77-96; 118-139; 150-163; 200-208; 217-237; 252-271

SOSUI 28-50; 75-97; 113-135; 148-170; 192-214; 221-243; 251-273

DAS-TMfilter 25-53; 74-98; 116-140; 149-165; 195-209; 224-237; 251-273

TOP-PRED 26-46; 80-100; 123-143; 148-168; 195-215; 221-241; 250-270

TMHMM 2.0 27-49; 78-100; 113-135; 150-167; 219-241; 251-273

Phobius 26-56; 76-100; 112-135; 147-167; 195-217; 223-241; 253-274

PDB code: 2PNO_A

Observed 5-33; 43-74; 75-100; 104-147

MemBrain 6-21; 59-77; 80-92; 108-132

THUMBU 10-24; 61-90; 109-135

SOSUI 5-27; 64-86; 116-138

DAS-TMfilter 7-23; 61-75; 77-89; 111-140

TOP-PRED 5-25; 58-78; 114-134

TMHMM 2.0 7-29; 67-89; 109-131

Phobius 67-89; 110-131

PDB code: 2Q7M_A

Observed 10-37; 48-77; 81-101; 116-138

MemBrain 7-25; 63-79; 84-97; 112-138

THUMBU 10-21; 67-86; 116-143

SOSUI 5-27; 67-89; 118-140

DAS-TMfilter 8-21; 67-78; 84-92; 116-139

TOP-PRED 5-25; 63-83; 118-138; 141-161

TMHMM 2.0 7-26; 71-93; 118-140

Phobius 7-26; 69-93; 114-137; 143-160

PDB code: 2QTS_A

Observed 19-45; 401-433

MemBrain 19-41; 410-431

THUMBU 23-42; 411-426

SOSUI 20-42

DAS-TMfilter 23-36; 416-425

TOP-PRED 19-39; 233-253; 408-428

TMHMM 2.0 20-42

Phobius 20-38
